# Efficacy of Essential Oils of *Thymus vulgaris* and *Origanum vulgare* on *Echinococcus granulosus*

**DOI:** 10.1155/2014/693289

**Published:** 2014-08-10

**Authors:** P. E. Pensel, M. A. Maggiore, L. B. Gende, M. J. Eguaras, M. G. Denegri, M. C. Elissondo

**Affiliations:** ^1^Laboratorio de Zoonosis Parasitarias, Departamento de Biología, Facultad de Ciencias Exactas y Naturales, Universidad Nacional de Mar del Plata (UNMdP), Funes 3350, 7600 Mar del Plata, Argentina; ^2^Consejo Nacional de Investigaciones Científicas y Técnicas (CONICET), Avenida Rivadavia 1917, C1033AAJ Buenos Aires, Argentina; ^3^Laboratorio de Artrópodos, Departamento de Biología, Facultad de Ciencias Exactas y Naturales, Universidad Nacional de Mar del Plata (UNMdP), Funes 3350, 7600 Mar del Plata, Argentina

## Abstract

The aim of the present work was to determine the *in vitro* effect of *T. vulgaris* and *O. vulgare* essential oils against *E. granulosus* protoscoleces and cysts. Essential oils were added to the medium resulting in thymol final concentrations of 10 *μ*g/mL. The essential oils had a time-dependent effect provoking the complete loss of protoscolex viability after 72 days of postincubation. The results were confirmed at the ultrastructure level. Loss of infectivity in protoscoleces incubated with *O. vulgare* after 60 days was observed. On the other hand, the weight of cysts recorded in mice inoculated with *T. vulgaris* treated protoscoleces was significantly lower than that obtained in control group. Gamma-glutamyl-transpeptidase activity was readily detected in the culture supernatant of protoscoleces treated either with the essential oils or thymol. *T. vulgaris* and *O. vulgare* essential oils and thymol can induce cell apoptosis of protoscoleces after short incubation times. The efficacy of *T. vulgaris* and *O. vulgare* essential oils was also demonstrated *in vitro* on *E. granulosus* murine cysts. Our data suggest that essential oils of *T. vulgaris* and *O. vulgare* have anthelmintic effect against protoscoleces and cysts of *E. granulosus*.

## 1. Introduction

Hydatidosis or human cystic echinococcosis (CE) is a cosmopolitan zoonotic disease that can be found in people and livestock infected with the larval stage of the tapeworm parasite* Echinococcus granulosus*. Dogs are the primary definitive hosts for this parasite, with livestock and humans acting as intermediate hosts. The outcome of infection is cyst development in the liver, lungs, or other organ systems [1].

Depending on different factors such as cyst type, size, location, and presence/absence of complications, the options for treatment of CE are as follows: surgery, PAIR (puncture, aspiration, injection of protoscolicidal agent, and reaspiration), antiparasitic drug treatment, or watch and wait [2]. The evidence supporting any of the four treatment modalities, from carefully designed clinical studies, is insufficient, and choosing treatment options for patients remains controversial [3].

For inoperable cases, chemotherapy with the benzimidazole-methylcarbamate (BZ) compounds as albendazole (ABZ) and mebendazole remains the only alternative. Approximately a third of patients treated with BZ drugs have been cured and 30–50% of CE-patients have demonstrated significant regression of cyst size. However, 20–40% of cases do not respond favourably [4]. Moreover, adverse reactions against BZ under long-term chemotherapy include elevation of transaminases, alopecia, gastrointestinal disturbances, and leucopenia [5]. Praziquantel (PZQ), a heterocyclic pyrazinoisoquinoline derivative, has been proposed to be used alongside BZ in CE-patients. Nevertheless, further studies are required to determine whether there are significant benefits from combination therapy with ABZ and PZQ over monotherapy with ABZ [6]. With regard to these difficulties, the development of a new therapeutic drug for human treatment of cystic echinococcosis is necessary.

A problem that has emerged with the use of synthetic anthelmintics is the development of parasitic resistance, which can threaten the success of treatment in humans [7–12]. Consequently, the search of new therapeutic alternatives such as the use of traditional medicinal plants has been increased [13].

The pharmaceutical properties of aromatic plants are partially attributed to essential oils [14]. Essential oils are volatile, natural, and complex compounds characterized by a strong odour and formed by aromatic plants as secondary metabolites. These substances may be constituted for about 20 to 60 components at quite different concentrations. Therefore, the biological effects of essential oils may be the result of a synergism of all molecules or reflect only those of the main molecules present at the highest levels according to gas chromatographical analysis [15]. On the other hand, there are studies where the principal component of essential oil was more active on its own than when present in essential oil which suggests that antagonism may have occurred between components of the oil [16].

Because of its hydrophobic character, the essential oils or their components present a great potential for pharmacological applications as antimicrobial agents [14, 17–20]. The role of essential oils against parasitic helminths has been studied by several authors [13, 21–23]. However, there are few publications about the effect of these substances on* E. granulosus*. The* in vitro* effect of the essential oils of* Rosmarinus officinalis* (rosemary),* Mentha pulegium*,* M. piperita*,* Pistacia khinjuk* (pistachio), and* Trachyspermum ammi* (ajowan) was demonstrated against protoscoleces of* E. granulosus* [24–27]. Moreover, the* in vitro* effect of thymol was shown against protoscoleces, microcysts, and cysts of* E. granulosus* [28, 29].

Apoptosis is a form of cell death which is triggered by external factors and ultimately leads to the cell's self-destruction [30]. The induction of apoptosis was demonstrated for several essential oils [31–33]. On the other hand, Paredes et al. [34] proposed that apoptosis may be a cellular mechanism underlying hydatid cyst infertility, which is observed in some herbivores and humans. Moreover, drug-induced apoptosis in* E. granulosus* protoscoleces has been reported [30, 35].

*Thymus vulgaris* (thyme) and* Origanum vulgare* (oregano) are shrubs distributed in areas of the Mediterranean and Asia, both members of family Lamiaceae.* In vitro*, thyme and oregano essential oils have significant antibacterial, antifungal, and antiparasitic activities [17, 37–39]. The biological activity of these substances has been related to their phenolic compounds content such as thymol and carvacrol, which represent between 40 and 50% of oils [40]. The aim of the present work was to determine the* in vitro* effect of* T. vulgaris* and* O. vulgare* essential oils against* E*.* granulosus* protoscoleces and cysts.

## 2. Materials and Methods

### 2.1. Plant Material and Oil Extraction

Oregano and thyme leaves, completely formed, were collected separately in Sierra de la Ventana (38°9′ S, 61°48′ W), Buenos Aires Province, Argentina, between December and February. Specimens were classified and stored in the herbarium of the Faculty of Exact and Natural Sciences, Universidad Nacional de Mar del Plata. Fresh plant material was dried prior to distillation (20–27°C and ~50% RH); and dried leave oils were obtained by hydrodistillation using a Clevenger-type apparatus [41] for 2 h. An average of 100 g of leaves was used in each experiment, and several distillations were performed until the volume required to run all trials was reached. The oils were dried over anhydrous sodium sulphate and stored in screw-capped dark glass vials at 5°–8°C until further testing.

### 2.2. Essential Oil Analyses

Thin layer chromatography (TLC) of the essential oils was performed on silica gel plates (0.2 mm Kieselgel 60 F254, Merck). Both oils were applied to two TLC plates using an aliquot of 5 *μ*L (using Drummond microcapillaries) and developed (93 : 7 toluene/ethyl acetate). Separated compounds were sprayed with sulphuric acid in ethanol, and later on with vanillin in ethanol, followed by heating at 110°C [42].

Essential oil composition was elucidated by gas chromatography (GC) and gas chromatography-mass spectrometry (GC-MS), using a Shimadzu GC-17A chromatograph equipped with a flame ionization detector (FID). Separations were performed using a DB-1 fused silica column (60 m × 0.248 mm, film thickness 0.25 mm). Oven temperature was programmed from 60 to 240°C at 3°C/min and the final temperature was held for 10 min. Injector and detector temperatures were set at 230°C and 250°C, respectively, and carrier gas N at a flow of 0.9 mL/min. The GC/MS analysis was performed on a Perkin- Elmer, QMass 910 GC operating at 70 eV, equipped with a DB-5 fused silica column (30 m × 0.25 mm, film thickness 1.0 mm). The injector and detector temperatures were 250°C; oven temperature was programmed from 60°C (5 min), 60 to 220°C at 3°C/min, and 220°C (8 min) and carrier gas He at a flow of 1 mL/min. The identification of EO's components was based on the comparison of their mass spectra with those reported in literature [43, 44]. Quantitative data were obtained by integration of FID area percents without the use of collection factors.

### 2.3. Physicochemical Properties Determinations

The yields of the essential oils were calculated according to Retamar [45]. Density to 20°C, triplicate of 1 mL of essential oil was weighed and the average of the values obtained was calculated [46]. The refractive index was determined at 20°C ± 0.05°C with an Abbe refractometer, in compliance with AOAC official method 921.08 [47].

Oils were subjected to ultraviolet-visible (UV-vis) spectroscopy at a concentration of 200 ppm to oregano and 12,5 ppm to thyme [46], using a Shimadzu UV-2101PC scanning spectrophotometer.

The infrared spectroscopy (IR) of the samples was recorded as a thin liquid film on NaCl windows with FTIR Mattson, model Genesis II spectrophotometer. The spectra were accumulated from 8 scans measured with a resolution of 2 cm^−1^ in the range of 500–4500 cm^−1^.

### 2.4. *In Vitro* Culture of* E. granulosus* Protoscoleces and Drug Treatment

Hydatid cysts from liver and lungs of naturally infected cattle were obtained from an abattoir located in the southeast of the Buenos Aires province, Argentina. Protoscoleces were removed from cysts under aseptic conditions and washed several times with phosphate-buffered saline (PBS, pH 7.2). Viability was assessed by the methylene blue exclusion test [48]. Viable and free protoscoleces (2000 per Leightont tube) were cultured in 10 mL of medium 199 (Gibco), containing 60 *μ*g/mL penicillin, 100 *μ*g/mL streptomycin, 50 *μ*g/mL gentamicin, and 4 mg/mL glucose.

Essential oils of* T. vulgaris* and* O. vulgare* were dissolved in medium 199 using propylene glycol (PG) as an emulsifier and were added to the medium resulting in thymol final concentrations of 10 *μ*g/mL (according to thymol concentration on these essentials oils determined by CG). Thymol (Sigma) was dissolved in dimethyl sulphoxide (DMSO) at a drug concentration of 10 mg/mL and was added to the medium resulting in final concentrations of 10 *μ*g/mL. Protoscoleces incubated with culture medium alone and with culture medium containing PG or DMSO were used as controls. Each experiment was repeated five times.

Culture tubes were followed microscopically every day. Samples of protoscoleces (approximately 90–100 protoscoleces in 180 *μ*L of incubation medium) from each treatment and the controls were taken every 5 to 6 days for viability assessment.

### 2.5. Determination of Infectivity to Mice

Animal procedures and management protocols were carried out in accordance with the 2011 revised form of* The Guide for the Care and Use of Laboratory Animals* published by the US National Institutes of Health. Unnecessary animal suffering was avoided throughout the study.

Samples of protoscoleces that had been incubated for 60 days in the presence of thymol and essential oils of* T. vulgaris *and* O. vulgare* were rinsed in medium 199. After sedimentation, they were resuspended in medium 199 supplemented with 60 *μ*g/mL penicillin, 100 *μ*g/mL streptomycin, and 50 *μ*g/mL gentamicin and the concentration was adjusted to 3000 protoscoleces/mL. Of this protoscoleces suspension resulting from the* in vitro* treatments (*T. vulgaris, O. vulgare*, thymol, and PG control), 0.5 mL was inoculated into each of four female CF1 mice (body weight 25 ± 5 g) by intraperitoneal injection. At 5 months after infection, mice were euthanized, necropsied, and examined for larval growth.

### 2.6. Enzyme Assay

Gamma-glutamyl-transpeptidase (GGT) was proposed as an ideal viability marker during* in vitro* pharmacological studies against* E. granulosus* protoscoleces [49]. Enzyme assay was carried out in sterile tissue culture plate (Nunc, 96 wells). Protoscoleces (1500 per well) were cultured under aseptic conditions in 0.2 mL of medium 199 per well supplemented with antibiotics and glucose.* In vitro* incubations were performed at 37°C without changes of medium. Enzyme activity was determined from culture protoscolex supernatants following the procedure described by Cumino et al. [49]. The activity was measured at 37°C in a recording Shimadzu model UV-vis spectrophotometer, the volume of the reaction mixture was 1 mL with 50 *μ*L of enzyme samples, and measurements were made after 30 min of incubation. Gamma-glutamyl-transpeptidase (GGT) was determined following the Szasz method [50], whereas the rate of increase in absorbance is due to release of p-nitroaniline. The substrate solution is an aqueous buffered solution containing 2.9 mM L-gamma-glutamylp-nitroanilide, 100 mM glycylglycine, and 5 mM MgCl_2_. Measurements were performed in triplicate.

### 2.7. TUNEL Assay

A commercially available kit (Apop Tag Plus In Situ Apoptosis Detection Kit S7101; Chemicon International a Serologicals Company; USA and Canada) was used to detect the 3′-OH ends of the DNA strands according to manufacturer's instruction. Briefly, protoscoleces from different treated groups were fixed in Karnovsky's solution (paraformaldehyde 2% (w/v), glutaraldehyde 2.5% (v/v), and 0.05 M cacodylate pH 7.2) at 4°C for 48 h and then embedded in paraffin. Tissue sections (5 mm) were deparaffinized and pretreated with Proteinase-K solution (20 *μ*g/mL) at room temperature for 15 min. The endogenous peroxidase activity was quenched using 3% (v/v) hydrogen peroxide in phosphate-buffered saline (PBS) at room temperature. Sections were incubated in a mixture of terminal deoxynucleotidyl transferase and digoxigenin-labeled dideoxynucleotide in a humidified chamber at 37°C for 1 hour. After reacting with a stop buffer, the sections were incubated with an antidigoxigenin peroxidase conjugate for 30 minutes. Peroxidase activity was detected by exposing the sections to a solution containing 3.3′-diaminobenzidine tetrahydrochloride (DAB). Sections were then counter-stained with hematoxylin. Negative controls were treated with distilled water in place of the terminal deoxynucleotidyl transferase enzyme. After color development, slides were observed under light microscope. Nuclei of apoptotic cells were stained brown with TUNEL reagents; normal nuclei had no brown staining but showeda blue color with hematoxylin.

### 2.8. Mouse Infection and Procedures for* In Vitro* Incubation of Cysts

Female CF-1 mice (body weight 25 g ± 5) were infected by intraperitoneal inoculation with 1,500* E. granulosus *protoscoleces/animal, suspended in 0.5 mL of medium 199 (Gibco). Animals were housed in a temperature-controlled (22 ± 1°C), light-cycled (12-hour light/dark cycle) room. Food and water were given* ad libitum*.

At 8 months after infection, mice with experimental secondary CE were euthanized and necropsy was carried out immediately thereafter. At necropsy, the peritoneal cavity was opened and the hydatid cysts were carefully removed [51].

Groups of 5 cysts were placed in Leighton tubes containing 10 mL of medium 199. Thymol and the essential oils of* T. vulgaris* and* O. vulgare* were added to the medium resulting in thymol final concentrations of 10 *μ*g/mL. Cultures were maintained at 37°C without changes of medium during the entire drug incubation period [52]. Culture tubes were followed macro- and microscopically every day. Samples of cysts from each of the dosing groups and the controls were taken and then fixed for electron microscopy. The criteria for cysts viability assessment included the loss of turgidity, the collapse of cysts, and the ultrastructural observation of the germinal layer as described by Elissondo et al. [52].

### 2.9. Statistical Analysis

Log-rank test was used to assess the survival differences of protoscoleces after exposure to thymol and essential oils of* T. vulgaris* and* O. vulgare*. For infectivity studies, differences in mean weight of cyst recovered from mice inoculated with treated and untreated protoscoleces were tested using the Mann-Whitney *U* nonparametric test (Wilcoxon rank sum test). All statistical analyses were performed with the software BioEstat 5.0 [53]. *P* values less than 0.05 were considered to be statistically significant.

### 2.10. Electron Microscopy

Samples of protoscoleces and cysts cultured* in vitro* were processed for scanning and transmission electron microscopy (SEM and TEM) as described by Elissondo et al. [48, 52].

## 3. Results

### 3.1. Essential Oils Analyses

The chemical composition of oregano and thyme essential oils obtained by hydrodistillation was determined by GC-MS.  Table 1 shows the main chemicals present in both essential oils. Aromatic compounds (thymol and carvacrol) dominated the composition.

**Figure 1:**
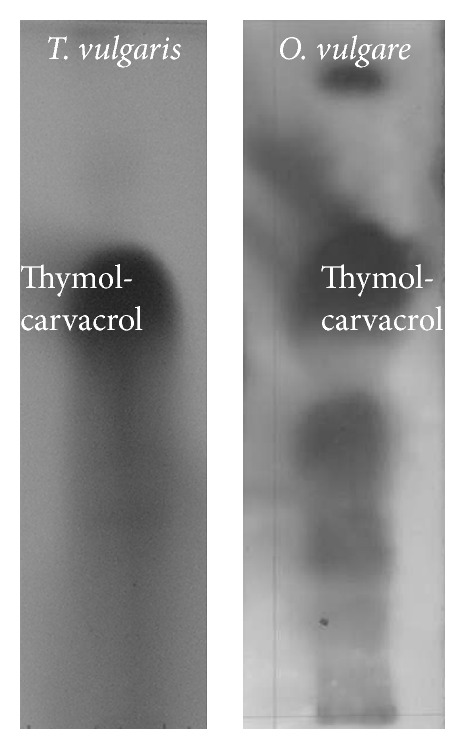
Thin layer chromatography of* T. vulgaris *and* O. vulgare* essential oils.

The main compounds and the Rfs values determined by TLC of the essential oils extracted from the different plants studied are shown in Figure 1.* O. vulgare *showed 5 bands, being the more intense the superior one to an Rf of 0.60 corresponding to the mixture of thymol and carvacrol.* T. vulgaris *showed two bands, being able to identify a pink band at Rf 0.64 and another less intense blue band at a lower Rf. Major compounds identified by TLC were consistent with data obtained from GC-ES analysis.

Physicochemical properties of the essential oils are shown in Table 2.

**Figure 2:**
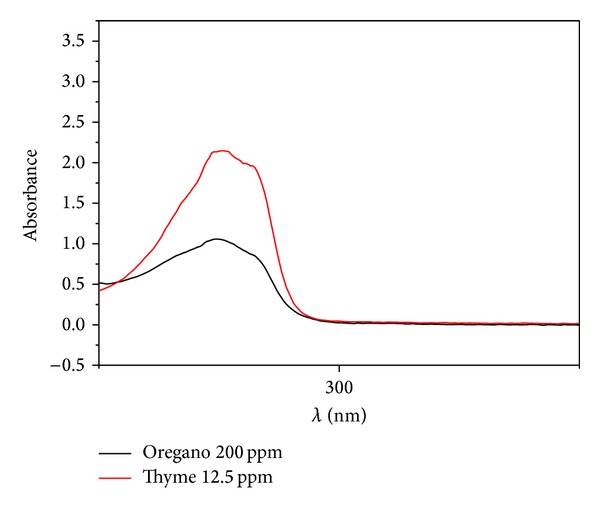
UV comparative spectra of* O. vulgare *and* T. vulgaris* essential oils.

**Figure 3:**
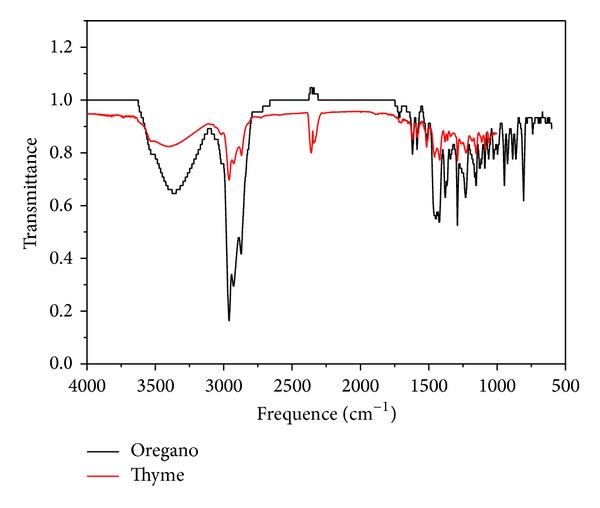
IR comparative spectra of* O. vulgare *and* T. vulgaris* essential oils.

No difference in the UV absorption curves of* O. vulgare* in relation to* T. vulgaris* essential oils was observed; both showed a peak absorbance at 275 nm but in the case of thyme oil, this peak was more pronounced (Figure 2). On infrared spectra, it could be observed that both oils presented similar IR patterns (Figure 3).

### 3.2. *In Vitro* Protoscoleces Incubation

**Figure 4:**
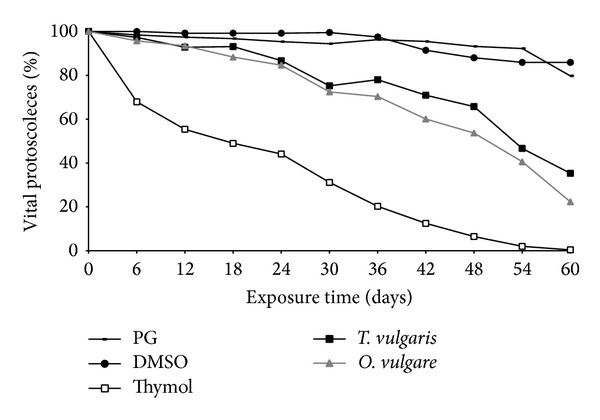
Survival of* E. granulosus* protoscoleces after exposure to thymol and essential oils of* T. vulgaris *and* O. vulgare*. Each point represents the mean percentage of vital protoscoleces from five different experiments. (PG: propylene glycol, DMSO: dimethyl sulphoxide).

The survival of* E. granulosus* protoscoleces after exposure to* T. vulgaris* and* O. vulgare* essential oils and thymol is shown in Figure 4.

Control protoscoleces cultured in medium 199 + PG or in medium 199 + DMSO remained viable (92.2 ± 2.4% and 85.9 ± 2.1%, resp.) after 60 days of incubation (Figure 4). Essential oil of* T. vulgaris* provoked a protoscolicidal effect, significantly reducing (*P* < 0.01) the viability of protoscoleces to 35.3 ± 2.8% after 60 days of incubation (Figure 4). Essential oil of* O. vulgare* significantly reduced (*P* < 0.01) the viability of protoscoleces to 22.3 ± 1.2% after 60 days (Figure 4). There was no significant difference (*P* > 0.05) between the protoscolicidal effect of essential oils of* T. vulgaris* and* O. vulgare*. Viability was 0% at day 72 for both essential oils (data not shown). On the other hand, the loss of viability of the protoscoleces incubated with thymol became clearer after 12 days of postincubation (p.i.), where the percentage value was 55.4 ± 8.2%, reaching 0% after approximately 60 days (Figure 4).

**Figure 5:**
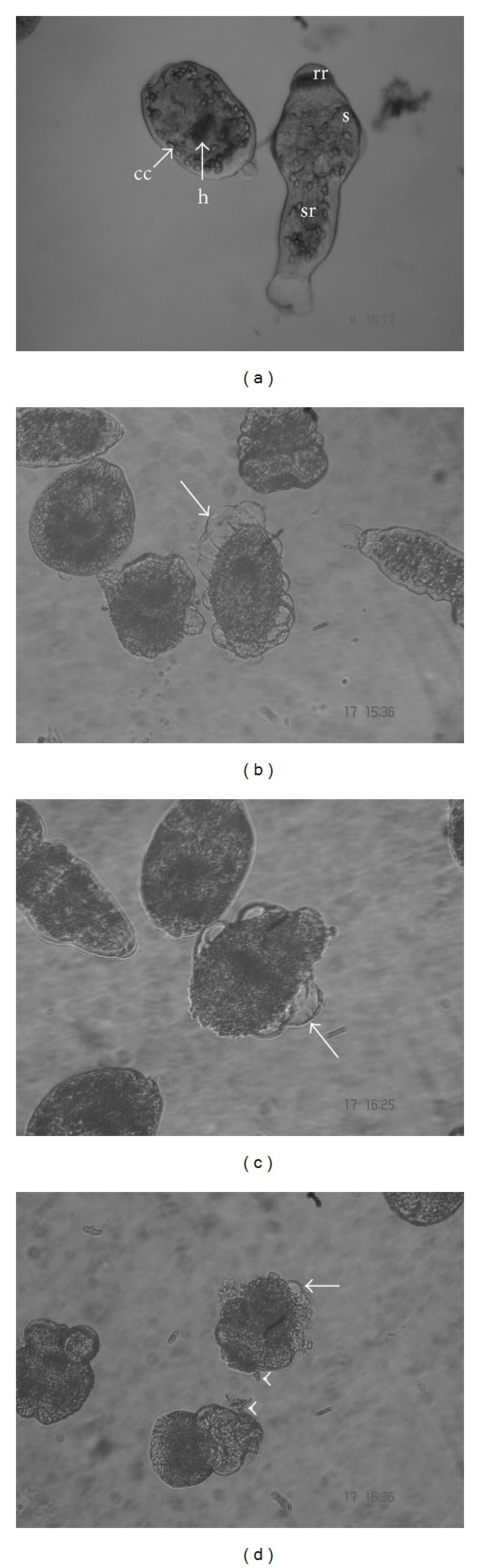
Light microscopy of* E. granulosus* protoscoleces incubated* in vitro* with thymol and essential oils of* T. vulgaris* and* O. vulgare*. (a) Control protoscoleces (cc: calcareous corpuscles, h: hooks, rr: rostellar region, s: suckers, and sr: soma region; 3 days p.i., 500x). (b)-(c) Note the presence of numerous blebs in the tegument of treated protoscoleces (arrows). (b)* T. vulgaris *(3 days p.i., 400x). (c)* O. vulgare* (3 days p.i., 500x). (d) Protoscoleces incubated with thymol during 2 days. The tegument was markedly altered (arrow). Note the loss of hooks (arrowheads, 400x).

**Figure 6:**
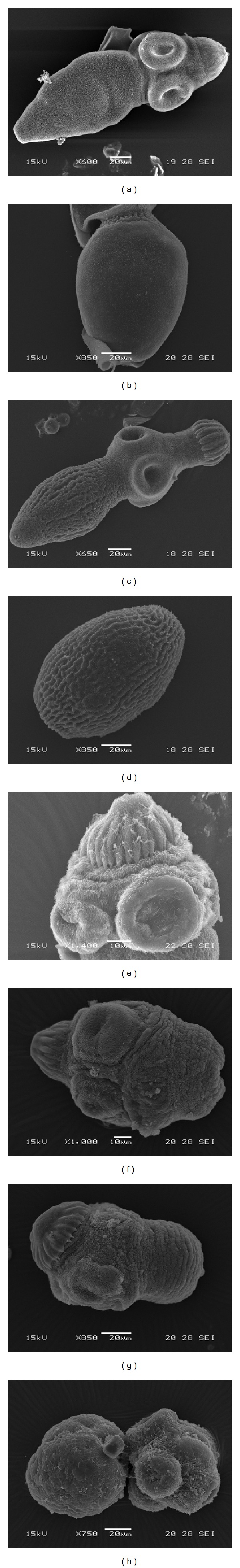
Scanning electron microscopy of* E. granulosus* protoscoleces incubated* in vitro* with thymol and essential oils of* T. vulgaris* and* O. vulgare*. (a) Evaginated control protoscolex (6 days p.i., 600x). (b) Invaginated control protoscolex (36 days p.i., 950x). (c) Altered protoscolex after 6 days p.i with* T. vulgaris*. Note the loss of microtriches at rostellar region (650x). (d) Invaginated altered protoscolex incubated with* O. vulgare* during 6 days (850x). (e) Scolex region of an evaginated protoscolex after 6 days with thymol. The shedding of microtriches can be observed (1400x). (f) Evaginated protoscolex (*T. vulgaris*, 36 days p.i.). Note the shedding of microtriches at the scolex region, the presence of numerous blebs, and the contraction of the soma region (1000x). (g) Altered and contracted soma region (*O. vulgare*, 36 days p.i.). Note the rostellar disorganization and shedding of microtriches (850x). (h) Completely altered protoscoleces (thymol, 24 days p.i., 900x).

The results of the viability tests coincide with tissue damage observed at the structural and ultrastructural level. Control protoscoleces revealed no changes in structure and ultrastructure throughout the experimental period (Figures 5(a), 6(a), and 6(b)). After 3 days p.i., the presence of numerous blebs in the tegument and contraction of soma region of essential oil treated protoscoleces was observed by inverted microscope (Figures 5(b) and 5(c)). Other alterations such as rostellar disorganization and loss of hooks could be seen after 6 days of incubation. The same alterations could be observed between 1 and 2 days p.i. in thymol treated protoscoleces (Figure 5(d)).

Studies by SEM revealed that ultrastructural damage was produced in drug-treated protoscoleces (Figure 6). After 6 days p.i., alterations in the tegument and contraction of soma region were observed in essential oil treated protoscoleces. Moreover, rostellar disorganisation and shedding of microtriches of the rostellar region were detected (Figures 6(c) and 6(e)). At 36 days p.i., loss of morphology was evidenced (Figures 6(d) and 6(f)). Thymol produced similar ultrastructural effects to those observed with the essential oils. After 6 days p.i., markedly tegumental alterations were detected (Figure 6(g)). At 24 days p.i., protoscoleces were altered with complete loss of morphology (Figure 6(h)).

### 3.3. Determination of Infectivity to Mice

Control protoscoleces developed an average of 6 ± 2.6 g of cysts, similar to the inoculation of freshly isolated protoscoleces. This result showed that the infectivity of control protoscoleces was not affected during* in vitro* incubation for 60 days. Loss of infectivity in protoscoleces incubated with thymol and* O. vulgare* after 60 days was observed, since all of the protoscoleces failed to develop into cysts following their inoculation into mice. Protoscoleces incubated with* T. vulgaris* developed an average of 0.29 ± 0.4 g of cysts. However, the weight of cysts recorded in mice inoculated with* T. vulgaris* treated protoscoleces was significantly lower (*P* < 0.05) than that obtained in the control group.

### 3.4. Determination of GGT Enzymatic Activity of Protoscoleces Treated with Thymol and Essential Oils of* T. vulgaris* and* O. vulgare* Drugs

**Figure 7:**
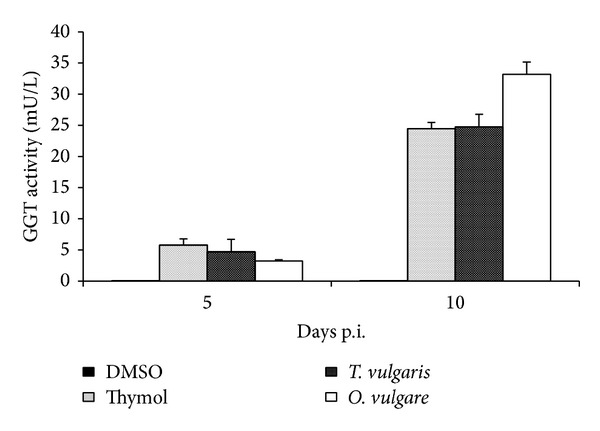
GGT activity detected in the supernatant of* in vitro* cultures of* E. granulosus* protoscoleces exposed to thymol and essential oils of* T. vulgaris* and* O. vulgare*.

GGT activity in culture supernatants of protoscoleces treated with thymol and essential oils of* T. vulgaris* and* O. vulgare* is shown in Figure 7. GGT activity was barely detectable in control supernatants throughout the experimental period. In contrast, the activity of GGT was detected in culture supernatants of drug treated protoscoleces at 5 days p.i. Enhancement of enzyme activity upon treatment with drugs was time dependent. Interestingly, the viability of essential oil treated protoscoleces at similar incubation time was slightly decreased (Figure 4).

### 3.5. TUNEL Assay

**Figure 8:**
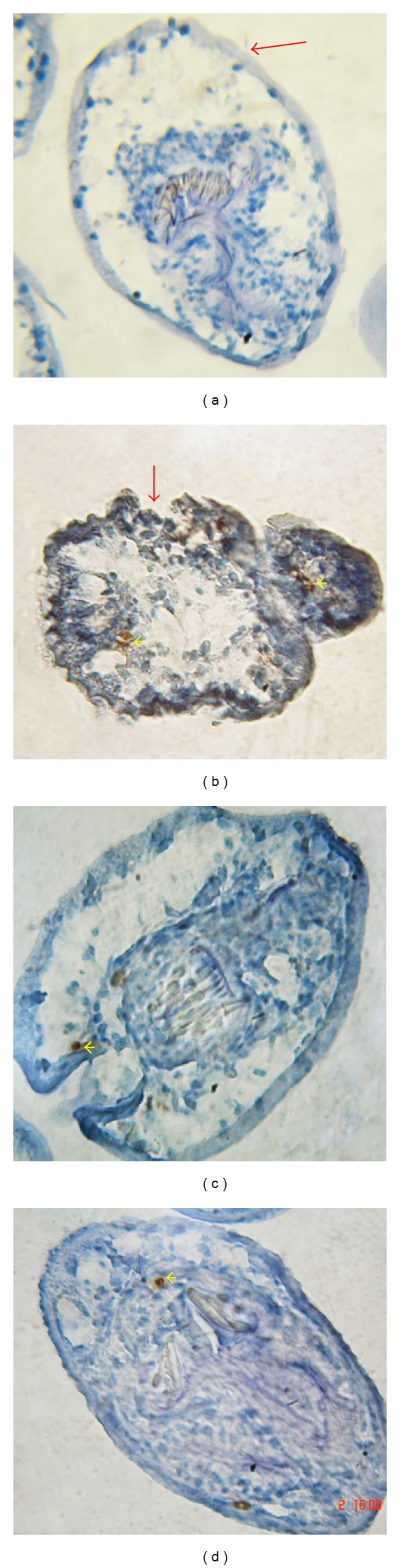
Light micrographs of TUNEL staining of* E. granulosus* protoscoleces incubated* in vitro* with thymol and essential oils of* T. vulgaris* and* O. vulgare*. (a) Invaginated control protoscolex (16 hours p.i., 1280x). Note the integrity of the tegument (red arrow). (b)–(d) Protoscoleces showing TUNEL-positive nuclei (yellow arrowheads). (b) Evaginated protoscolex (thymol, 8 h p.i., 1700x). Note the presence of numerous apoptotic nuclei and the loss of the integrity of the tegument (red arrow). (c) Invaginated protoscolex with the presence of few TUNEL-positive nuclei (*T. vulgaris*, 16 h p.i., ×1500). (d) Protoscolex incubated with* O. vulgare *during 16 h (×1500). Few TUNEL-positive nuclei could be observed.

Figure 8 shows representative TUNEL images of protoscoleces treated with thymol and essential oils of* T. vulgaris *and* O. vulgare*. Cells from control sections (Figure 8(a)) showed no apoptotic nuclei. The results of TUNEL assay clearly reveal presence of* in situ* DNA fragmentation in the nuclei of treated protoscolex tissue. Apoptosis in cells of thymol treated protoscoleces was detected at 8 h p.i., while in essential oil treated parasites it was observed after 16 h p.i. (Figures 8(b)–8(d)). In addition, protoscoleces with loss of the integrity of the tegument (Figures 8(b)–8(d)) showed higher levels of apoptotic nuclei than protoscoleces with intact tegument (Figure 8(a)).

### 3.6. *In Vitro* Cysts Incubation

All control cysts appeared turgid with no observable collapse of the germinal layer over the course of the* in vitro* experiment. In contrast, a loss of turgidity was detected in cyst treated with* T. vulgaris* and* O. vulgare *essential oils and thymol (Table 3).

**Figure 9:**
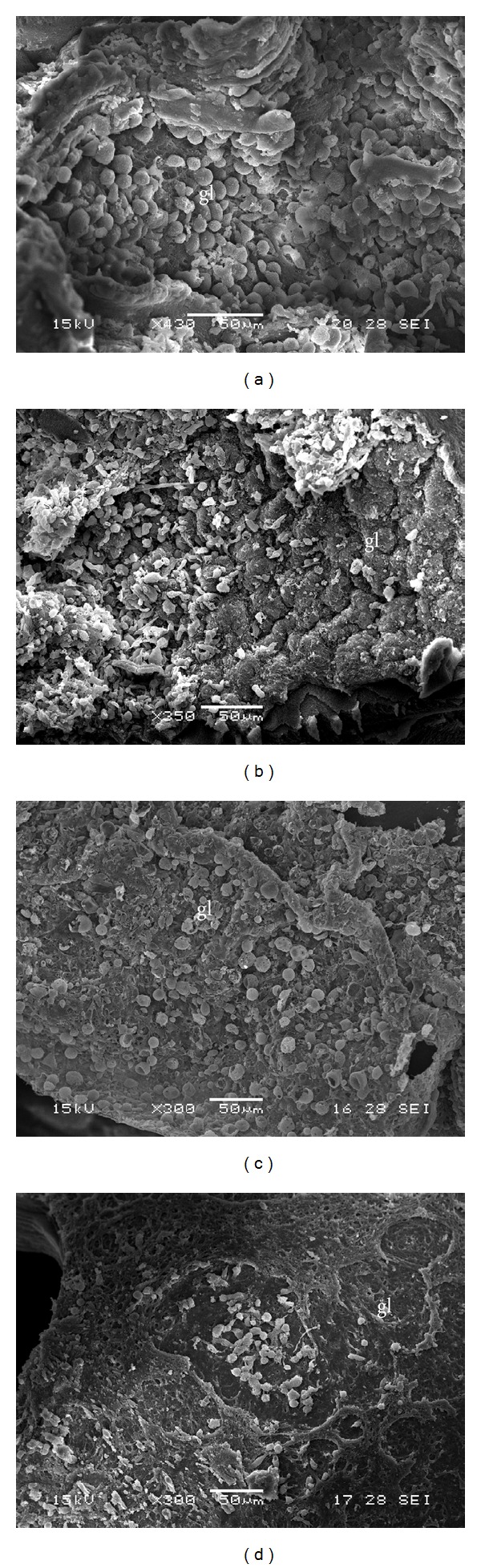
Scanning electron microscopy of* E. granulosus *murine cysts incubated* in vitro* with thymol and essential oils of* T. vulgaris* and* O. vulgare*. (a) Control murine cyst with an intact germinal layer (6 days p.i., gl: germinal layer; 430x). (b) Cyst incubated with* T. vulgaris* during 6 days (350x). The germinal layer is altered. (c) Cyst exposed to* O. vulgare* after 6 days p.i. (500x). (d) Extensive damage of the germinal layer (thymol, 3 days p.i., 300x). Only cellular debris could be observed.

Control cultures exhibit no ultrastructural alterations in parasite tissue during the whole incubation period (Figure 9(a)). Studies by SEM revealed that the germinal layer of treated cysts lost the feature multicellular structure (Figures 9(b)–9(d)).

## 4. Discussion

The present work describes for the first time the* in vitro* efficacy of* T. vulgaris* and* O. vulgare* essential oils against protoscoleces and cysts of* E. granulosus*. Essential oils of* T. vulgaris* and* O. vulgare* had a time-dependent effect provoking the loss of protoscolex viability. Moreover, protoscoleces treated with* O. vulgare *and thymol lost their infectivity, since following inoculation of treated parasites in mice no cysts could be recovered after five months of postinfection.

The results were confirmed at the ultrastructure level by SEM. The alterations included contraction of soma, blebs formation in tegument, rostellar disorganisation, and loss of hooks and microtriches. These changes have been observed in* E. granulosus *protoscoleces following* in vitro* treatment with other essential oils or their components [24, 26, 28, 29]. Moreover, the same alterations have also been reported by other authors working with synthetic drugs like BZ [48, 54], praziquantel [55], ivermectin [51, 56], and nitasoxanide [57].

GGT activity in culture supernatants of protoscoleces treated with* T. vulgaris* and* O. vulgare* essential oils and thymol was also measured as a marker of tegumental damage. GGT activity was readily detected into the culture supernatant of protoscoleces treated either with essential oils and thymol. Moreover, our observations are in accordance with those reported by Cumino et al. [49]. The high sensitivity and accuracy of the enzymatic method was demonstrated in relation to ultrastructural studies and determination of viability by the methylene blue exclusion test. The release of the enzyme precedes the tegumental damage.

The efficacy of* T. vulgaris* and* O. vulgare* essential oils and thymol was also demonstrated* in vitro *on* E. granulosus *murine cysts. SEM studies revealed that the germinal layer of cysts lost the characteristic multicellular structure, clearly showing disintegrated areas. The ultrastructural changes were analogous to those reported by other authors [29, 52, 57, 58].

*T. vulgaris *essential oil showed thymol as a main compound in their composition while the predominant components in* O. vulgare *essential oil were thymol and carvacrol. We suggest that the main components found in the essential oils assayed could be responsible for the markedly anthelmintic effect detected. Despite the differences observed in their chemical composition (see Table 1), the essential oils assayed had a similar anthelmintic activity. Data about the essential oil composition obtained with CG MS and TLC are in accordance with those reported by Wagner et al. [42], Bagamboula et al. [59], and Gende [36]. Physicochemical properties were analyzed with the aim of establishing the quality, purity, and chemical stability of the essential oil. All physicochemical properties obtained ranged within the prospective values, if compared with the results by Montes [46] and Retamar [45].

In the present study, thymol had a slightly higher effect against* E. granulosus* metacestodes than did the essential oils assayed. However, there are studies where the biological activity of the essential oils was superior to thymol alone. Santoro et al. [17] have shown that trypomastigotes are more sensitive to* T. vulgaris* essential oil in relation to purified thymol. The discrepancies observed could be explained by the differences of their qualitative and quantitative composition. The composition of essential oils can differ according to harvesting seasons, geographical sources, age, and vegetative cycle stage [60–62]. Therefore, in order to obtain essential oils of constant composition, they have to be extracted under the same conditions from the same organ of the plant [15].

On the other hand, the inherent activity of the essential oils can be expected to relate to the proportions in which the components are present and to interactions between them [63]. Thus, the observed anthelmintic activity of* T. vulgaris* and* O. vulgare* could be explained by the possible antagonic interactions between the components present in these essential oils. Antagonic effect between components was also described by Liolios et al. [64] working with other essential oils. Antimicrobial activity of* O. dictamnus* was lower than thymol and carvacrol acting alone. Botelho et al. [65] have suggested that the lower antibacterial of* Lippia sidoides* essential oil when compared to thymol and carvacrol may be attributed to an antagonistic effect of the minor components on the activity of the essential oil.

The mechanism of action of essential oils and their components has not been studied in great detail. Since the essential oils are complex mixtures of several compounds, their antimicrobial activity is not attributable to one specific mechanism but there are several targets in the cell [66, 67].

In eukaryotic cells, essential oils can provoke depolarisation of the mitochondrial membranes by decreasing the membrane potential [68–70]. Essential oils change the fluidity of membranes, which become abnormally permeable resulting in leakage of radicals, cytochrome C, calcium ions, and proteins, as in the case of oxidative stress and bioenergetic failure. Permeabilization of outer and inner mitochondrial membranes leads to cell death by apoptosis and necrosis [71, 72]. Apoptosis induced by thymol in cancer cells was associated with mitochondrial pathway [73, 74]. TUNEL assay revealed that thymol and essential oils of* T. vulgaris* and* O. vulgare* can induce apoptosis in the cells of protoscoleces. The induction of apoptosis was detected after short incubation times. As it was mentioned, drug-induced apoptosis in* E. granulosus* protoscoleces has also been reported by other authors [30, 35].

In conclusion, our data suggest that essential oils of* T. vulgaris* and* O. vulgare* have an anthelmintic effect against protoscoleces and cysts of* E. granulosus*. Thymol had a considerably greater effect than that observed with essential oils. However, the GGT activity measured in culture supernatants of protoscoleces treated with thymol or essential oil was similar.

Further* in vitro* and* in vivo* bioassays will be required to fully evaluate the potential of these essential oils or some of their components as useful alternatives for the treatment of hydatid disease. Moreover, the evaluation of the* in vitro* effects of carvacrol alone and in combination with thymol is in progress and the results of this study could be a promising option for CE chemotherapy.

## Figures and Tables

**Table 1:** Quantitative composition of *O. vulgare* and *T. vulgaris *essential oils.

	Component	%
*O. vulgare *	Thymol	19.71
	Carvacrol	20.14
	*γ*-Terpinene	12.77

*T. vulgaris *	Thymol	65.4
	Carvacrol	5.4
	Borneol	0.7
	Bornyl acetate	0.1

Ref: thyme essential oil data were analyzed for this study, while the composition of oregano oil was extracted from Gende 2009 [36].

**Table 2:** Physicochemical properties of the essential oils: yield v/w %, density to 20°C (*ρ*_20_), and refraction indexes (*n*_*D*_^20^).

Physicochemical properties	Essential oils	
	*O. vulgare *	*T. vulgaris *
Yield v/w %	0.4–1.6%	1.0–1.3%
*ρ*_20_ (g/mL)	0.932	0.923
*n*_*D*_^20^	1.488	1.504

**Table 3:** Time of appearance (days p.i.) of different indicators of tissue damage on *E. granulosus *murine cysts, after their incubation with thymol and *T. vulgaris * and * O. vulgare* essential oils, under *in vitro* conditions.

Parameters of the study	Postincubation days			
	Thymol 10 *µ*g/mL			
	Thymol	*T. vulgaris *	*O. vulgare *	Control
Loss of cyst turgidity	2	3	3	—

Appearance of collapsed cysts	3	5	5	—
